# Supplementary figures and images for: Mimicking the Liver Sinusoidal Endothelial Cell Niche In Vitro to Enhance Fenestration in a Genetic Model of Systemic Inflammation
Source: Cells. 2025 Apr 21;14(8):621. doi: 10.3390/cells14080621 (PMC12025456; doi:10.3390/cells14080621)

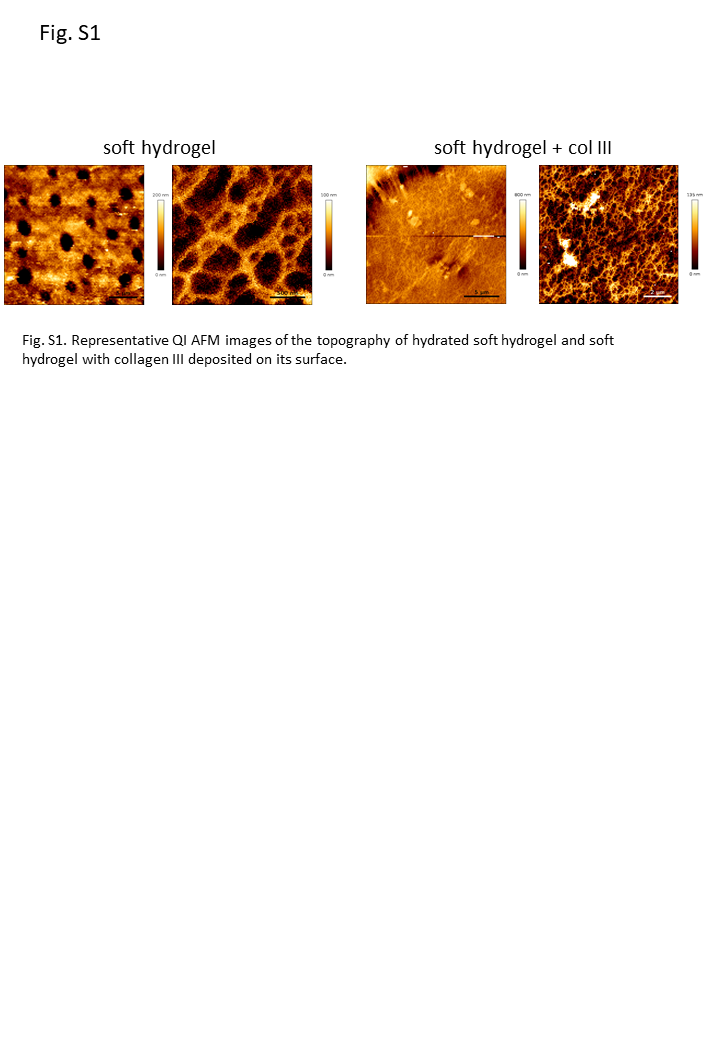

Supplement: Supplementary file 1 [file cells-14-00621-s001.zip › suppl Figures gels_v3/Fig. S1.PNG]

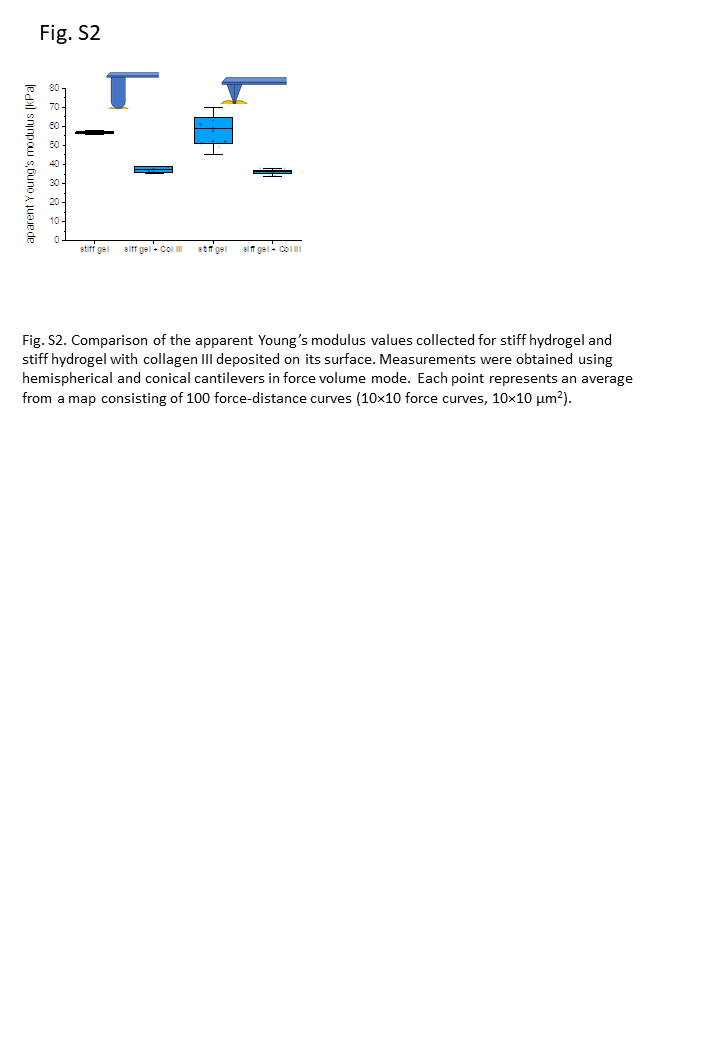

Supplement: Supplementary file 1 [file cells-14-00621-s001.zip › suppl Figures gels_v3/Fig. S2.PNG]

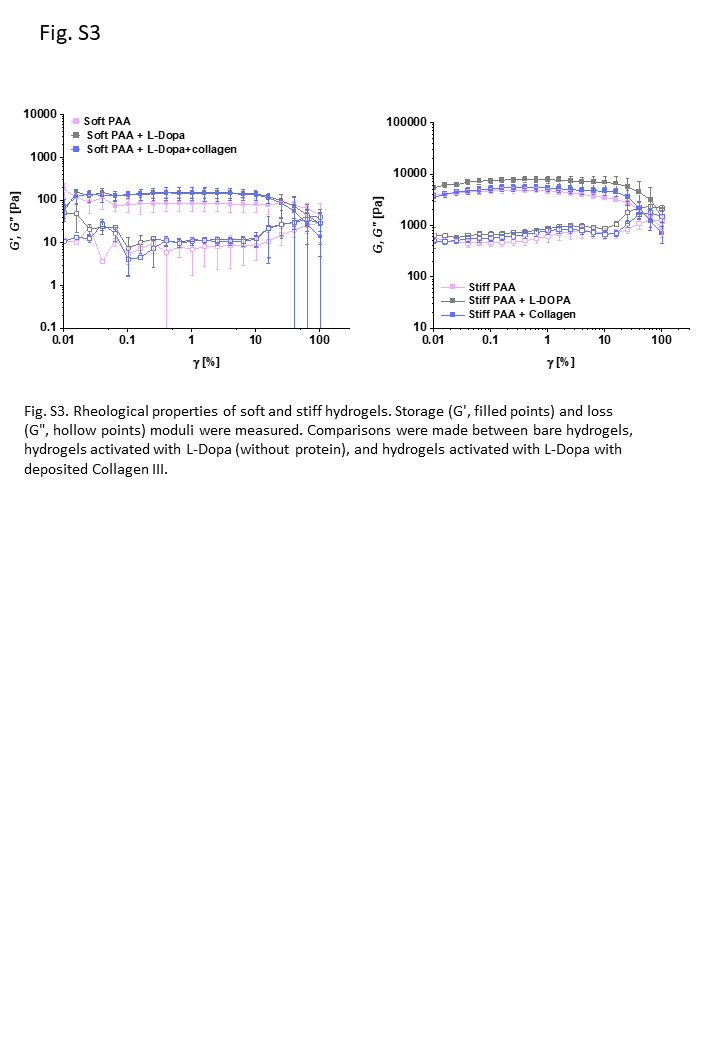

Supplement: Supplementary file 1 [file cells-14-00621-s001.zip › suppl Figures gels_v3/Fig. S3.PNG]

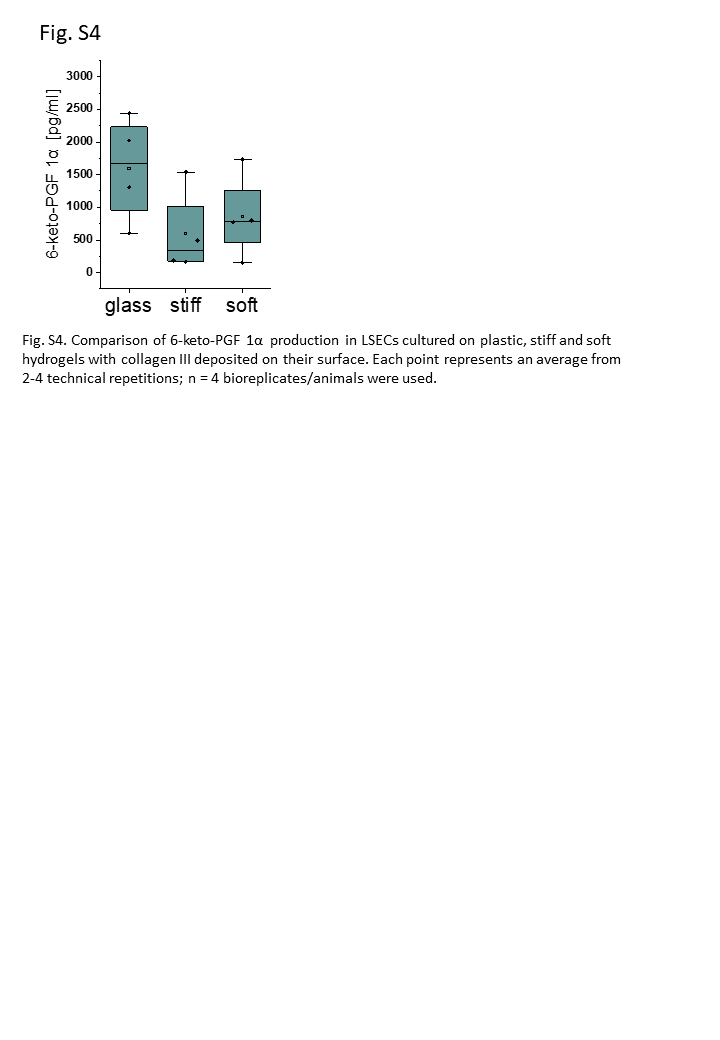

Supplement: Supplementary file 1 [file cells-14-00621-s001.zip › suppl Figures gels_v3/Fig. S4.PNG]

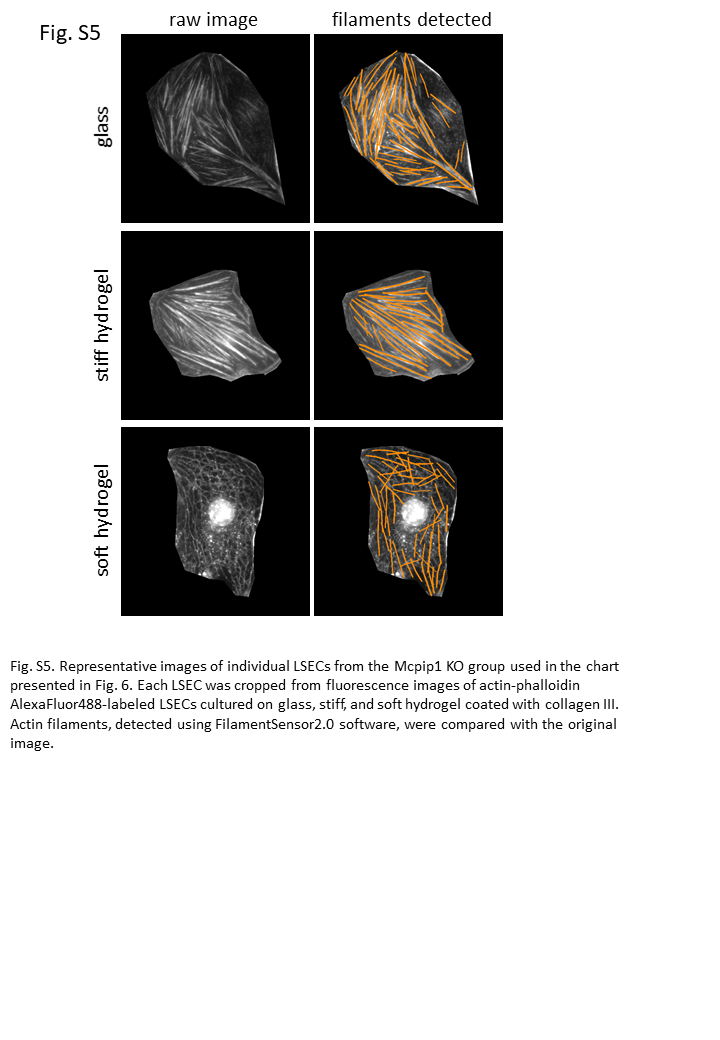

Supplement: Supplementary file 1 [file cells-14-00621-s001.zip › suppl Figures gels_v3/Fig. S5.PNG]

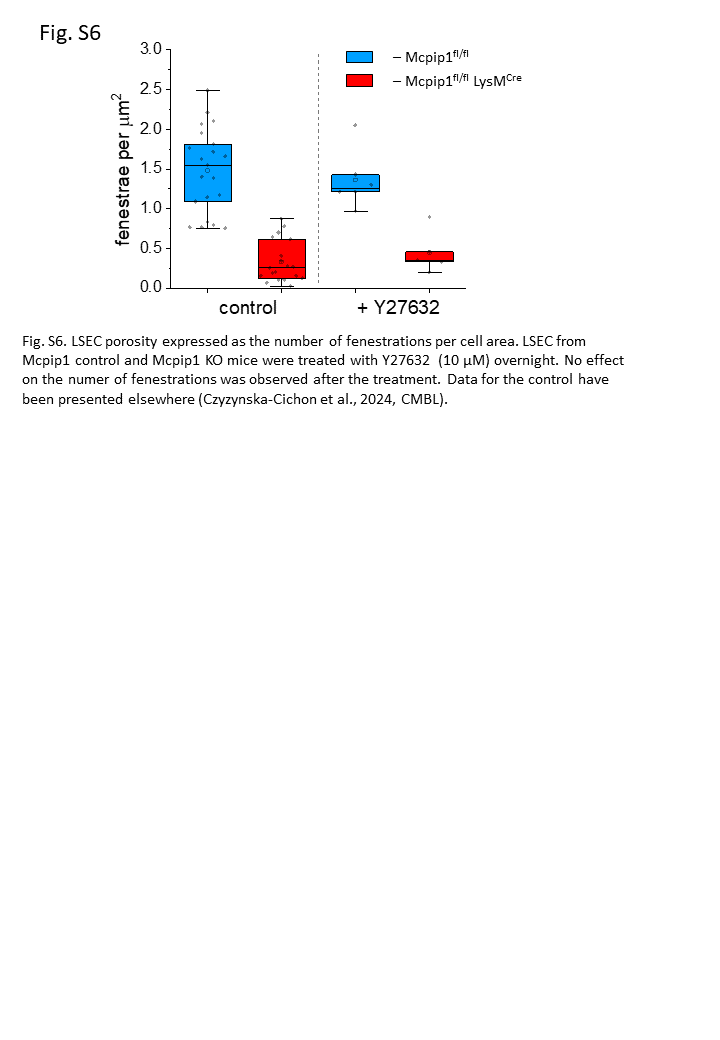

Supplement: Supplementary file 1 [file cells-14-00621-s001.zip › suppl Figures gels_v3/Fig. S6.PNG]
